# A Canadian Critical Care Trials Group project in collaboration with the international forum for acute care trialists - Collaborative H1N1 Adjuvant Treatment pilot trial (CHAT): study protocol and design of a randomized controlled trial

**DOI:** 10.1186/1745-6215-12-70

**Published:** 2011-03-09

**Authors:** Karen EA Burns, Clarence Chant, Orla Smith, Brian Cuthbertson, Robert Fowler, Deborah J Cook, Peter Kruger, Steve Webb, Jamal Alhashemi, Guillermo Dominguez-Cherit, Carlos Zala, Gordon D Rubenfeld, John C Marshall

**Affiliations:** 1 Interdepartmental Division of Critical Care Medicine and Departments of Pharmacy, University of Toronto, Toronto, Ontario, Canada; 2 Keenan Research Centre and the Li Ka Shing Knowledge Institute, St. Michael's Hospital, Toronto, Ontario, Canada; 3 Department of Critical Care Medicine, Sunnybrook Health Sciences Centre, Toronto, Ontario, Canada; 4 Department of Clinical Epidemiology and Biostatistics, McMaster University, Hamilton, Ontario, Canada; 5 Intensive Care Unit, Princess Alexandra Hospital, Brisbane, Queensland, Australia; 6 Intensive Care, Royal Perth Hospital, Perth, Western Australia, Australia; 7 King Abdulaziz University Hospital, Jeddah, Saudi Arabia; 8 Instituto Nacional de Ciencias Medicas y Nutricion "Salvador Zubiran", Mexico City, Mexico; 9 Hospital Central de San Isidro, Dr. Melchor Angel Posse, San Isidro, Buenos Aires, Argentina

## Abstract

**Background:**

Swine origin influenza A/H1N1 infection (H1N1) emerged in early 2009 and rapidly spread to humans. For most infected individuals, symptoms were mild and self-limited; however, a small number developed a more severe clinical syndrome characterized by profound respiratory failure with hospital mortality ranging from 10 to 30%. While supportive care and neuraminidase inhibitors are the main treatment for influenza, data from observational and interventional studies suggest that the course of influenza can be favorably influenced by agents not classically considered as influenza treatments. Multiple observational studies have suggested that HMGCoA reductase inhibitors (statins) can exert a class effect in attenuating inflammation. The Collaborative H1N1 Adjuvant Treatment (CHAT) Pilot Trial sought to investigate the feasibility of conducting a trial during a global pandemic in critically ill patients with H1N1 with the goal of informing the design of a larger trial powered to determine impact of statins on important outcomes.

**Methods/Design:**

A multi-national, pilot randomized controlled trial (RCT) of once daily enteral rosuvastatin versus matched placebo administered for 14 days for the treatment of critically ill patients with suspected, probable or confirmed H1N1 infection. We propose to randomize 80 critically ill adults with a moderate to high index of suspicion for H1N1 infection who require mechanical ventilation and have received antiviral therapy for ≤ 72 hours. Site investigators, research coordinators and clinical pharmacists will be blinded to treatment assignment. Only research pharmacy staff will be aware of treatment assignment. We propose several approaches to informed consent including a priori consent from the substitute decision maker (SDM), waived and deferred consent. The primary outcome of the CHAT trial is the proportion of eligible patients enrolled in the study. Secondary outcomes will evaluate adherence to medication administration regimens, the proportion of primary and secondary endpoints collected, the number of patients receiving open-label statins, consent withdrawals and the effect of approved consent models on recruitment rates.

**Discussion:**

Several aspects of study design including the need to include central randomization, preserve allocation concealment, ensure study blinding compare to a matched placebo and the use novel consent models pose challenges to investigators conducting pandemic research. Moreover, study implementation requires that trial design be pragmatic and initiated in a short time period amidst uncertainty regarding the scope and duration of the pandemic.

**Trial Registration Number:**

ISRCTN45190901

## Background

### Influenza and Swine Origin Influenza A/H1N1 Infection (H1N1)

On June 11, 2009, the World Health Organization (WHO) declared that infection with the Swine Origin Influenza A/H1N1 virus had reached pandemic proportions [1]. Cases were recorded in more than 180 countries and outbreaks that strained national resource capacities were documented in Canada, Australia, Chile, Argentina, and elsewhere.

Throughout history, pandemic influenza has posed a recurrent threat to human populations. Seasonal influenza is responsible for more than 50,000 deaths per year in the United States [2]. The capacity of the influenza virus to mutate and spread from animals to humans has resulted in intermittent pandemics. The 1918 pandemic was the largest in recent history and caused between 40 and 50 million deaths worldwide [3]. Smaller pandemics in 1957 and 1968 were associated with mortality spikes but their effects were mild at the population level [4]. Experts believe further pandemics will certainly occur, but are uncertain about when. Several years ago, the avian H5N1 influenza virus threatened to be the vector of the next pandemic. While highly virulent when transmitted from infected chickens to humans, the absence of human-to-human transmission resulted in a small number of cases worldwide [5].

In early 2009, a novel strain of influenza, swine origin influenza A/H1N1 infection (H1N1) emerged in swine and rapidly spread to humans [6]. Originating in Mexico, the strain proved highly infectious and was spread by person-to-person contact with a predilection for younger hosts. While early epidemiologic data suggested that although H1N1 was highly infectious, it was less virulent [7,8] than anticipated with a case fatality rate of approximately 0.5% of infected individuals. For the majority of infected individuals, symptoms were mild and self-limited; however, a small percentage of infected individuals developed profound respiratory failure requiring extraordinary means of oxygenation support including high frequency oscillation (HFO) ventilation and extracorporeal membrane oxygenation (ECMO) [9]. Caring for the most severely ill patients during a pandemic results in an increased need for intensive care unit (ICU) resources and strains available personnel and equipment. There is little excess capacity to care for critically ill patients in most developed countries, and minimal capacity in developing countries.

### Rationale for Study Intervention

Supportive care and antiviral agents, especially neuraminidase inhibitors (such as oseltamivir and zanamivir), are the mainstay of treatment for influenza. While efficacious in reducing viral load and abating symptoms in ambulatory patients, their effects on outcomes in critically ill patients have not been established. Their utility in treating severe pandemic H1N1 influenza may be compromised by widespread use and emergence of viral resistance [10,11], limited supplies, policies regarding treatment strategies, and cost and availability, especially in developing countries [12]. Human and animal data suggest that the course of influenza may be favorably influenced by certain agents not classically considered as treatments for influenza [13-16] that are comparatively inexpensive and readily available. Such agents may provide independent benefit in treating viral infection and are attractive as adjuvant treatments.

### Statin Therapy for Influenza

Statins lower plasma lipid levels by inhibiting HMG CoA reductase, the enzyme responsible for converting HMG CoA to mevalonate, a rate-limiting step in cholesterol biosynthesis. Multiple observational studies have suggested that statins may be of benefit in patients with a variety of severe infections [17-22] by exerting an effect in attenuating inflammation [23,24]. The mechanism of this activity is uncertain but may involve their ability to restrict cholesterol availability in cell membranes of the innate immune system. Cholesterol is a key component of lipid rafts, membrane-associated microdomains that support cell signaling in response to exogenous inflammatory stimuli. Raft disruption may attenuate the cellular response to inflammatory stimuli [25,26].

Experimental studies show that pre-treatment with statins attenuates the severity of acute lung injury (ALI) following intestinal ischemia-reperfusion [27] and the inflammatory response to intravenous lipopolysaccharide challenge in human volunteers [28]. The combination of a statin and caffeine inhibits viral replication and attenuates lung injury in murine influenza models [29]. Population-based studies suggest that statins are associated with reduced inflammatory morbidity in critically ill patients receiving them [20] and reduced mortality in patients with influenza and chronic obstructive pulmonary disease (COPD) patients [30]. Reviewing 3,921 hospitalized patients with laboratory confirmed influenza, of whom 1,019 were receiving a statin at the time of admission, Vandermeer and colleagues found that statin use was independently associated with a reduced risk of death (adjusted OR = 0.34, 95% CI: 0.16-0.70) in a multivariable logistic regression model [31]. The benefits of statins appear to be best established in patients receiving them prior to the onset of infection. Cohort studies report conflicting results on the ability of statins to attenuate organ dysfunction with Schmidt and colleagues finding that statins reduce mortality in critically ill patients with multiple organ dysfunction syndrome [32] and Kor et al reporting that statin use was not beneficial in resolving organ dysfunction [33].

### Rosuvastatin: Risks and Benefits

Statins are widely used and well-tolerated medications. Rosuvastatin differs from other HMG CoA reductase inhibitors in that only up to 10% of the parent compound is metabolized by cytochrome P450 2C9 and 2C19 and not by P450 3A4 [34]. As a result, it exhibits fewer drug-drug interactions, and serum concentrations are not affected by CYP2D6 gene polymorphism. Adverse events seen with rosuvastatin are generally mild and may include muscle symptoms, however, myopathy and rhabdomyolysis occur infrequently in patients taking 40 mg/day or less. As with other HMG-CoA reductase inhibitors, a dose-related increase in liver transaminases and creatinine kinase (CK) has been observed in small numbers of patients taking rosuvastatin. The overall occurrence of clinically significant transaminase increases is low (< 1%) and similar across rosuvastatin doses ranging from 5 mg/day to 40 mg/day [35]. Patients with severe liver disease may have increased exposure to rosuvastatin [35].

### Study Objective

The International Forum of Acute Care Trialists (InFACT) is an informal alliance of investigator-led clinical trials networks whose remit is to improve the care of critically ill patients through the promotion of scientifically rigorous clinical research. Based on our preliminary understanding of H1N1 influenza, members of the Canadian Critical Care Trials Group (CCCTG), working in collaboration with members of InFACT, designed the Collaborative H1N1 Adjuvant Treatment (CHAT) Pilot Trial to investigate the feasibility of conducting an international therapeutic trial during a global pandemic and the potential for adjuvant rosuvastatin, in addition to standard treatment, to influence clinical outcomes in influenza A (H1N1) associated critical illness.

## Methods

### Study Design

**Figure 1:**
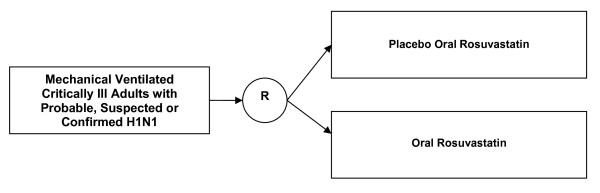
**Study Overview**.

A multi-national feasibility RCT involving adult ICUs in Canada, Saudi Arabia, Mexico, Argentina Australia/New Zealand. An overview of the study design is provided in Figure 1 (**see **Figure 1).

### Primary Objective

[1] The ability to recruit the desired patient population under pandemic conditions (i.e., the proportion of eligible patients enrolled in the CHAT Pilot Trial).

### Secondary Objectives

[2] Adherence to the medication administration regimen as outlined in the study protocol.

[3] The ability to collect the required primary and secondary endpoints for the planned full CHAT trial.

[4] The number of patients who receive open-label statins.

[5] The number of consent withdrawals.

[6] The impact of approved consent models on recruitment rates.

### Patient Screening

A dedicated research coordinator will screen patients for eligibility on a daily basis in the ICUs at participating sites. If the study inclusion criteria are fulfilled and no exclusion criteria are present, the research coordinator will identify the patient as a potential study participant. The attending physician or intensivist will confirm eligibility for participation in the CHAT Pilot RCT.

### Inclusion Criteria

Criteria to identify potential candidates for study inclusion will include:

1) Critically ill adult patients ≥ 16 years of age admitted to an adult ICU for any reason with suspected, probable or confirmed novel swine origin influenza A/H1N1 infection (**see **Additional File 1).

2) Requiring mechanical ventilation (invasive or non-invasive)

3) Receiving antiviral therapy (any medication at any dose and for any intended duration) for ≤ 72 hours

4) Attending physician or intensivist must have a 'moderate' to 'high' index of suspicion for H1N1.

### Exclusion Criteria

1) Age < 16 years

2) Do not resuscitate or re-intubate order documented on chart or anticipated withdrawal of life support

3) Weight < 40 kg

4) Unable to receive or unlikely to absorb enteral study drug (e.g., incomplete or complete bowel obstruction, intestinal ischemia, infarction, short bowel syndrome)

5) Rosuvastatin specific exclusions:

a. Already receiving a statin

b. Allergy or intolerance to statins.

c. Receiving niacin, fenofibrate, cyclosporine, gemfibrozil, any protease inhibitor (including but not limited to lopinavir and ritonavir) or planned use of oral contraceptives or estrogen therapy during the ICU stay.

d. CK exceeds 5,000 U/L **or **ALT exceeds 8 times the upper limit of normal (ULN).

6) Severe chronic liver disease (Child-Pugh Score 11-15) (**see **Additional File 2)

7) Previous enrolment in this trial

8) Pregnancy or breast feeding

9) At the time of enrolment, receipt of > 72 hours of antiviral therapy.

10) Known or suspected clinically significant myositis or myopathy.

### Eligible, Non-Randomized Patients

We will record reasons why eligible patients are not randomized into the CHAT Pilot Trial under the following categories:

a) Substitute Decision Maker (SDM) consent refusal

b) Physician refusal of consent

c) SDM not available to provide consent and waived/deferred consent not permitted

d) SDM does not exist and waived/deferred consent not permitted

e) Coordinator workload

f) Coordinator not available during eligible time-window

g) Enrolment in a competing trial

h) Other (specification required)

To enhance trial feasibility and given the need to conduct a pragmatic trial, co-enrolment in other prospective observational studies or RCTs (not investigating similar or alternative H1N1 treatments) in operation in the ICU setting will be permitted and recorded in sites where permitted by the local Research Ethics Boards (REBs) [36].

### Study Randomization

To preserve allocation concealment, participants will be randomized centrally. Randomization lists will be distributed by the study methods centre to the research pharmacies of participating centres. Stratified variable block randomization, based on centre alone, will be performed to take into consideration differences in patient characteristics at participating ICUs. Day one will be considered the day of study treatment initiation, which may or may not be the same day of randomization.

### Consent

A waiver of consent is the preferred option for participant enrollment given the context of a global pandemic. We have constructed a consent algorithm to direct the consent process at sites where the local REB has not approved a waiver of consent (**see **Additional File 3). In this trial, we will request that patients be enrolled in the study, and consent be deferred to SDMs or to the patient (whomever is able to provide consent first), when it is not possible to obtain consent within 24 hours. Consent may be obtained in person or by telephone as per local practices. In the event that patients die before providing consent, we request permission from REBs to include data collected during study participation.

### Study Treatment Overview

Using randomization lists provided by the study methods centre, research pharmacists will assign critically ill adults to once daily enteral administration of rosuvastatin or matched placebo for 14 days. Only the research pharmacy staff will be aware of the assigned treatment arm. The site investigators, research coordinator, clinical pharmacist involved in the care of the patient and all other study personnel will remain blinded to treatment assignment. An oral placebo for nasogastric administration, identical in appearance (colour and consistency matched) to crushed rosuvastatin, will be prepared by Pharmacy 1 (Toronto, Canada) and supplied to the study sites. All other aspects of patient management will be left to clinician discretion as per pragmatic trial design. The Applied Health Research Centre of the Keenan Research Centre and Li Ka Shing Knowledge Institute (St Michael's Hospital, Toronto, Ontario) will be the Study Methods Centre.

### Study Drug Administration

#### Delivery Route

Study drug will be administered once daily through an enteral feeding tube or orally if the patient is able to safely take oral medications. The type and placement of the enteral feeding tube (nasogastric, nasoenteric, percutaneous endoscopic gastrostomy, orogastric, oroenteric, etc.) will be at the discretion of the attending clinical team. The ability to safely take oral medications will be determined by the patient's primary care team.

#### Initial Dose

The first study drug dose (rosuvastatin or placebo) will be administered within 4 hours of randomization as a loading dose of 40 mg unless the subject is of Asian (Chinese, Korean, Japanese, Phillipino, Vietnamese, or Asian-Indian) descent, age <18 years or has serum creatinine greater than or equal to 248 umol/L (2.8 mg/dL) (see requirements for dose adjustments below).

#### Subsequent Doses

Thereafter doses of 20 mg will be administered at 22:00 hrs daily (+/- 4 hours) starting on the next calendar day (study day 2) as a maintenance dose. If the patient is of Asian descent, is <18 years, or serum creatinine is greater than or equal to 248 umol/L (2.8 mg/dL) dose adjustments will be required according to the dose adjustment algorithm (see requirements for dose adjustments below). Dose adjustment is only necessary for patients with renal impairment and not receiving dialysis. Once dialysis is started and serum creatinine remains elevated, dose adjustment is not required.

#### Missed Doses

If for any reason a maintenance dose is not administered at the intended time, it may be administered subsequently but not more than 12 hours after the intended time of administration. If greater than 12 hours has elapsed since the last scheduled dose, the patient will receive another loading dose, and then maintenance dosing will resume on the next calendar day as outlined above. A missed dose, for reasons other than outlined under medication discontinuation will be considered a protocol violation.

#### Dose Adjustments

The loading (40 mg) and daily maintenance (20 mg) doses will be reduced by 50% for patients:

a) who have at least one parent of Asian descent (loading 20 mg and daily 10 mg),

b) whose age < 18 years (loading 20 mg and daily 10 mg),

c) whose serum creatinine concentration is greater than or equal to 248 umol/L (2.8 mg/dL) who are not on renal replacement therapy (loading 20 mg and daily 10 mg), and by 75% for patients:

d) who have at least one parent of Asian descent and/or age < 18 years *and *serum creatinine is greater than or equal to 248 umol/L (2.8 mg/dL) (loading 10 mg and daily 5 mg).

#### Concomitant Medication Administration

Intermittent oral antacids should be administered no closer than 6 hours before or after administering rosuvastatin to avoid influencing study drug absorption [37]

#### Duration of Treatment

Given the substantial potential for false negative influenza results, we will continue adjuvant treatment administration, regardless of H1N1 testing results (positive or negative), for 14 days or until a criterion for cessation is met. If patients are liberated from mechanical ventilation (invasive or non-invasive) and discharged from the ICU between days 1 and 9 they will be advanced to day 10 of study drug administration. In the event that patients remain in the ICU for observation (e.g., possible reintubation or initiation of non-invasive ventilation) then study drug administration will NOT be advanced to day 10 until they are discharged.

### Completion of Study Drug Administration

Study drug administration will be stopped when one of the following conditions is met, whichever comes first:

1. 14 days after randomization

2. Hospital discharge (including transfer to an alternate care facility)

3. Death

4. CK noted to exceed 5,000 U/L (in the absence of an alternative diagnosis) or patient is determined to have clinical myositis or myopathy (at the discretion of the primary care team, patient may be re-challenged with study drug if CK or clinical findings no longer meet this criterion).

5. Alanine aminotransferase (ALT) exceeds 8 times the ULN (in the absence of an alternative diagnosis)

6. Co-administration of any of the following: niacin, fenofibrate, cyclosporine, gemfibrozil, lopinavir, ritonavir, oral contraceptives or estrogen

7. Attending physician or intensivist or SDM request to stop treatment.

We will request for data collection to continue in patients withdrawn from therapy prematurely. Clinicians will be encouraged to continue study medication despite negative H1N1 testing due to the potential for false negative results and the potential role for an anti-inflammatory agent (rosuvastatin) in severe lung disease. Decisions regarding continuation or discontinuation of antiviral treatment will be left to the discretion of the attending physician or intensivist.

### Study Co-interventions

It will not be feasible to protocolize ventilator and general clinical management under pandemic conditions. Adjunctive non-antibiotic, non-interventional management of sepsis, acute respiratory distress syndrome, and glycemic control will be at the discretion of the patient's primary clinicians. Key aspects of clinical management (such as choice of antiviral, dose of administration, duration of treatment, ventilator strategy, treatment with inhaled nitric oxide, HFO, prone positioning, ECMO, additional antiviral/anti-inflammatory treatments and treated episodes of infection) will be documented either as part of the Influenza A H1N1 (Swine Flu) ICU (Registry) Study for participating centres or on separate data forms for centres not participating in the registry. Antibiotic therapy may be prescribed for suspected or confirmed concomitant bacterial infection at the discretion of the attending physician.

### H1N1 Testing

Local testing procedures may be used to facilitate diagnosis of H1N1. Where no local testing procedures exist, we recommend using the following initial and repeat testing procedures. For all new admissions to adult ICUs meeting study inclusion criteria and having no exclusion criteria with non-confirmed H1N1 (i.e., all suspected or probable cases) or where uncertainty exists regarding prior testing, we request the following sequence of laboratory tests:

#### Initial Diagnostic Testing (assuming diagnosis not confirmed at ICU admission)

1. Paired deep nasopharyngeal (NP) swab AND either an endotracheal (ET) aspirate or bronchoalveolar (BAL) (or sputum if not intubated) for (a) influenza polymerase chain reaction (PCR) and (b) viral culture; and

2. Endotracheal aspirate (or sputum if not intubated) for gram stain, culture and sensitivity (C & S)

#### Repeat Testing (for patients with one positive test)

1. Repeat both tests (PCR and viral culture) from the site that was *positive *previously (i.e., ET or BAL or NP swab). If both ET and NP swab were *positive*, send repeat ET aspirate specimen at day 7 and at weekly intervals thereafter.

2. After the first negative specimen(s) from a previously positive site, send a repeat specimen from the same site at 48 hours after the first specimen was collected.

### Drug Level Specimen Collection

To verify adequate absorption of rosuvastatin, we will draw venous blood for peak and trough plasma rosuvastatin concentrations (total of 2 specimens) on day 7 (+/- 1 day). Day 7 will be the preferred day for trough and peak specimen collection. A trough level specimen will be drawn prior to day 7 (+/- 1 day) dose. A peak concentration specimen will be drawn 3 to 5 hours after the dose of study drug is administered. A maximum of 80 patients will have blood drawn for drug concentration analysis; however, only patients randomized to rosuvastatin will have their samples analyzed. Samples will be labeled and batched at the site, for shipment to St Michael's Hospital (Toronto, Canada) for analysis after the study is unblinded.

### Follow-Up

Research staff will assess participants daily for adverse effects for the duration of treatment. CHAT study participants will have daily CK and liver function [aspartate aminotransferase (AST) and ALT] levels collected as part of the study protocol. Study drug will be discontinued if hypersensitivity is suspected (see Criterion 7 Completion of Study Drug Administration).

We will record the number of patients receiving full treatment and reasons for the inability to complete the assigned treatment duration (i.e., death, transfer to an alternate care facility, study withdrawal, etc). The study Data Safety and Monitoring Board (DSMB) will review all patients withdrawn from the study, safety data, and deaths.

All patients will be followed until death or hospital discharge. We will record the vital status of all patients at 90 days and hospital discharge, whichever occurs first. At day 60 patients remaining on the ventilator will be deemed ventilator dependent. Randomized patients will be considered successfully extubated when they remain off positive pressure ventilation (invasive or non-invasive ventilation) for 48 consecutive hours. If patients are re-intubated *within *48 hrs following extubation, they will be followed until they achieve one of the aforementioned outcomes. Patients discharged from the ICU and requiring readmission and re-initiation of mechanical ventilation (invasive or non-invasive) will be treated according to usual practice and will not be randomized on a second occasion to this study.

### Study Outcomes

#### Primary Outcome

[1] Proportion of eligible patients enrolled in the CHAT pilot study.

#### Secondary Outcomes

[2] Adherence to the medication administration regimen as outlined in the study protocol.

[3] Proportion of completed primary and secondary endpoints for the planned full CHAT trial that are collected.

[4] Number of patients who receive open-label statins.

[5] Number of consent withdrawals.

[6] Recruitment rates by approved consent model.

### Sample Size Estimation (CHAT Pilot Trial)

Estimates are not available to allow precise sample size estimation of the primary outcome for the proposed CHAT pilot RCT. We propose to undertake a pilot study in a convenience sample of 80 patients with suspected, probable or confirmed H1N1 infection to assess trial feasibility.

### Data Collection

We will collect CHAT specific data starting at ICU admission using paper-based versions of the electronic data collection forms developed for the Influenza A H1N1 (Swine Flu) ICU (Registry) Study [7]. The forms will document baseline characteristics, enrolment into concurrent influenza research studies, co-morbidities, illness severity (**see **Additional File 4), vaccination status, co-interventions, feasibility outcomes and clinical outcomes for the planned definitive trial (primary: the proportion of patients successfully weaned from mechanical ventilation in less than 10 days; secondary: impact of rosuvastatin on ICU, 60 and 90 day, and hospital mortality and on ICU free days at day 60). In addition to the data forms developed for the Influenza A H1N1 (Swine Flu) ICU study, we developed 13 additional forms including an (i) eligibility and randomization form, (ii) severity of illness form, (iii) consent form, (iv) drug administration form, (v) H1N1 diagnostic test results form, (vi) laboratory data form, (vii) drug level (serum) specimen collection form, (viii) 60 day and 90 day outcomes form, (ix) comments and end of study investigator sign off, (x) protocol violation form: biochemistry, (xi) protocol violation form: medication administration/discontinuation, (xii) adverse event form, (xiii) serious adverse event form in randomized patients. For centres not participating in the registry, we drafted 10 additional forms to capture necessary demographic, treatment and outcomes information. In addition, we drafted a form to capture demographic data and outcomes on eligible but not randomized patients.

### Statistical Analysis

Descriptive statistics will be used to summarize the data. For univariate analyses, we will use the Chi-square test (alternatively, Fisher's exact test when the expected cell size is ≤ 5) and Student's t-test (alternatively, the Mann-Whitney U-test, if normality assumptions are not satisfied) for binary and continuous outcomes, respectively. All analyses will be conducted on an intention-to-treat basis.

Feasibility for the pilot study will be assessed by metrics that reflect our capacity to ultimately recruit a representative sample of 1,050 patients in the planned full CHAT trial. We will consider the study to be feasible if we recruit at least 30% (commonly used threshold in ICU studies) of all eligible patients in participating ICUs through careful review of site screening logs. Additionally, we expect that: (i) less than 10% of medication doses will fail to be administered in the absence of meeting one of the medication discontinuation criteria; (ii) less than 5% of data forms will be missing important primary and secondary outcomes data required for the planned full CHAT trial and (iii) no more than 10% of enrolled patients will be withdrawn prematurely due to open label use of statins or withdrawal of consent. We will describe recruitment rates based on approved consent models. Since centres in Australia and New Zealand will be permitted to use Atorvastatin and matching placebo (instead of Rosuvastatin/matching placebo), we propose to conduct the planned primary and secondary analyses (i) using the pooled data (rosuvastatin plus atorvastatin) and (ii) using rosuvastatin (as the predominantly used statin in the CHAT Trial) data alone.

A DSMB will oversee the trial and will consist of 3 individuals with expertise in viral infectious diseases, statistics and clinical critical care of which one will be international. The DSMB will hold a teleconference after either 30 patients have evaluable data or approximately 8 months after study initiation. Should the trial be completed, feasibility data from the pilot study will be analyzed by the DSMB at the end of the study. This information will be conveyed to the Steering Committee. Together the DSMB and Steering Committee will formulate a decision whether to proceed with the full trial. Clinical outcomes will remain blinded by study group assignment with a view to including them in the planned larger trial.

### Adverse Event Reporting

Investigators will evaluate any changes in laboratory values and physical signs and will determine if the change is clinically important and different from what is expected in the course of treatment of critically ill patients requiring mechanical ventilation for suspected, probable or confirmed influenza. If clinically important and unexpected adverse experiences occur, they will be recorded on an adverse event case report form. We will characterize adverse events (**see **Additional File 5) as expected, serious unexpected and study related or unanticipated.

### Other Considerations

We considered other factors (**see **Additional File 6) including patient withdrawals, consent (including telephone consent and waivers of consent) (**see **Additional Files 7 and 8), eligible non- randomized patients, equitable selection of subjects, justification for including vulnerable subjects, women of childbearing age, justification for excluding pregnant women, trial oversight and the trial data safety and monitoring board (**see **Additional File 6) in designing the CHAT Trial protocol. The investigators plan to make changes to the larger study protocol based on their experience in implementing the pilot trial. Regardless, we will publish the findings of the CHAT Pilot Trial, either alone or pooled with another trial evaluating the role of statins in a similar population, if recruitment ensues even if the study protocol is modified in important ways following conduct of the pilot trial or the planned larger trial never comes to fruition. Pilot trial data may also be combined with data from the larger trial if the latter trial comes to fruition, study personnel (including the data analyst) remain blinded to treatment assignment and no important modifications are made to the study protocol following the pilot trial.

## Discussion

Global concern arose from the threat of the H1N1 influenza pandemic. Despite the potential virulence of the illness, little is actually known about how severe disease develops or what treatments may confer benefit to critically ill patients. Even less is known about how to conduct clinical research in the setting of an evolving pandemic. Severe H1N1 infection primarily affects young and often previously healthy individuals. Early reports supported that aboriginal populations in Canada and Australia, obese individuals and women, especially pregnant women, appear to have a predilection for severe disease. Unlike the pandemic of 1918, the availability today of antiviral agents, antibiotics for secondary infection, and ICU supportive care interventions holds promise that the majority of patients with severe illness can be saved. The burden of severe H1N1 disease falls prominently on the ICU [8,38]. Consequently, the opportunity to learn about treatments for severe H1N1 disease and how to conduct pandemic critical care research rests within the ICU community.

Data from observational studies in humans and interventional studies in animals, suggests that the course of influenza may be favorably influenced by relatively inexpensive and readily available agents, such as rosuvastatin, that are not classically considered to be treatments for influenza. These agents are attractive as adjuvant treatments amidst emerging reports of oseltamivir resistance and threatened drug supply shortages. However, the ability to administer and test the efficacy of an adjuvant agent as a treatment for severe H1N1 infection remains to be established.

The CHAT pilot trial is designed to evaluate the feasibility of implementing a randomized controlled trial of adjuvant rosuvastatin for treating severe H1N1 infection under a pandemic. We aim to evaluate whether centres can adhere to the study treatment regimens, collect the required primary and secondary endpoints for the subsequent planned full CHAT trial, and document patients who receive open-label statins and consent withdrawals. We also seek to evaluate the impact of approved consent models on recruitment rates.

Several time-honored aspects of RCT design including the use of central randomization, preservation of allocation concealment, multi-level study blinding, and use of a matching placebo posed challenges to us in designing a pandemic protocol. We contemplated the necessity of including each of these study design features. After careful deliberation, we decided to include central randomization (using lists distributed by the study methods centre to participating centres), preserve multi-level blinding (by involving pharmacies at participating centres) and contract a local pharmaceutical company to prepare crushed drug and matching placebo (in the absence of industry supply of study drug and an available placebo). Recognizing that trial initiation may be delayed and recruitment curtailed if a priori in-person SDM consent was required, we considered use of alternative consent models. A priori in-person SDM consent not only hinges on the existence and availability of SDMs [39], but also the ability of SDMs to access hospitals during a pandemic.

Strengths of the proposed pilot trial design include the use of central randomization, allocation concealment, multi level blinding, standard criteria for medication discontinuation, and 90 day follow up. To ensure feasibility during a pandemic, we did not protocolize H1N1 testing, ventilator and sedation management or the clinical use of antibacterial agents. By merging study data with an Influenza Registry and capturing data on unique forms for centres not participating in the registry, we will, however, document key aspects of clinical management.

The conduct of an RCT during an evolving pandemic poses unique challenges not encountered during other forms of clinical research [40]. First, it is necessary to initiate studies quickly. The normal time interval from concept to first patient enrolment for a new RCT is typically of the order of two years or more. Second, the scope and duration of the pandemic is unknown and unpredictable. Third, the mitigating effects of large-scale vaccination programs and changes in H1N1 infectivity resulting from virus mutation are unknown. Fourth, the practicalities of conducting clinical research during a pandemic are unknown. For example, research personnel may be seconded to provide clinical care, pharmacists may face challenges in dispensing drug and placebo, REBs may not permit alternative consent models and it may be difficult to obtain consent from patients who may lack decision-making capacity and families, who may be unwell themselves, unable to visit the hospital or requested to stay away during the pandemic. Finally, faced with the clinical imperative of treating gravely ill and previously well, young patients, clinicians may opt to use open label treatment rather than permit enrolment into a blinded RCT. Because we believe that it is important to develop the capacity to initiate RCTs under pandemic conditions and to test study procedures prior to implementing a large scale RCT, we propose to conduct a multi-centre pilot trial to assess the feasibility of our clinical protocol and study procedures.

## Abbreviations

CHAT: Collaborative H1N1 Adjuvant Treatment; RCT: randomized controlled trial; ICU: intensive care unit; WHO: World Health Organization; HFO: high frequency oscillation; ECMO: extracorporeal membrane oxygenation; ALI: acute lung injury; COPD: chronic obstructive pulmonary disease; CK: creatinine kinase; InFACT: International Forum for Acute Care Trialists; CCCTG: Critical Care Trials Group; ULN: upper limit of normal; SDM: substitute decision maker; REB: Research Ethics Board; ALT: alanine aminotransferase; NP: nasopharngeal; ET: endotracheal tube; BAL: bronchoalveolar lavage; PCR: polymerase chain reaction; C & S: culture and sensitivity; AST: aspartate aminotransferase; TCPS: Tri-council Policy Statement; DSMB: data safety and monitoring board.

## Competing interests

The authors declare that they have no competing interests.

## Authors' contributions

All authors contributed to the conception and design of the CHAT Pilot Trial, drafted and revised the manuscript for important intellectual content. All authors approved the final version of the manuscript.

## Supplementary Material

Additional file 1 **Definitions for Diagnosis of H1N1 Infection**. File containing the definitions for Diagnosis of H1N1 Infection. Click here for file

Additional file 2 **Childs Pugh Classification**. File containing Childs Pugh classification. Click here for file

Additional file 3 **Consent Algorithm (if Waiver of Consent Not Used)**. File containing the consent algorithm (if waiver of consent not used). Click here for file

Additional file 4 **Illness Severity Assessment Scales**. File containing the illness severity assessment scales. Click here for file

Additional file 5 **Classification of Adverse Events**. File containing the classification of adverse events. Click here for file

Additional file 6 **Special Considerations**. File containing special considerations. Click here for file

Additional file 7 **Letter of Information**. File containing the letter of information. Click here for file

Additional file 8 **Telephone Consent**. File containing telephone consent. Click here for file
