# Supplementary material for: A Canadian Critical Care Trials Group project in collaboration with the international forum for acute care trialists - Collaborative H1N1 Adjuvant Treatment pilot trial (CHAT): study protocol and design of a randomized controlled trial
Source: Trials. 2011 Mar 9;12:70. doi: 10.1186/1745-6215-12-70 (PMC3068961; doi:10.1186/1745-6215-12-70)
Supplement: Additional file 1 — Definitions for Diagnosis of H1N1 Infection. File containing the definitions for Diagnosis of H1N1 Infection. [file 1745-6215-12-70-S1.DOC]

**Appendix 1: Diagnosis of H1N1 Infection**

**Adapted from the World Health Organization, the Centre for Disease Control, and the National Microbiology Laboratory^2^**

**Confirmed** – Laboratory confirmation of H1N1 flu virus infection with or without clinical symptoms by one or more of the following tests: reverse transcriptase (RT)-PCR, viral culture or a four-fold rise in H1N1 flu virus specific neutralizing antibodies.

**Probable** – Laboratory test positive for influenza A, untypeable with or without clinical symptoms.

**Suspected** – a person with acute febrile respiratory illness or recent history of fever with at least 2 of the following symptoms: cough, sore throat, rhinorrhea, limb/joint pain, or headache.
