# Supplementary material for: A Canadian Critical Care Trials Group project in collaboration with the international forum for acute care trialists - Collaborative H1N1 Adjuvant Treatment pilot trial (CHAT): study protocol and design of a randomized controlled trial
Source: Trials. 2011 Mar 9;12:70. doi: 10.1186/1745-6215-12-70 (PMC3068961; doi:10.1186/1745-6215-12-70)
Supplement: Additional file 2 — Childs Pugh Classification. File containing Childs Pugh classification. [file 1745-6215-12-70-S2.DOC]

**Appendix 2: Child Pugh Classification**

| **Measure** | **1 point** | **2 points** | **3 points** | **Units** |
| --- | --- | --- | --- | --- |
| **Bilirubin (Total)** | <34  (<2) | 34-50  (2-3) | >50  (>3) | μmol/l  (mg/dl) |
| **Serum Albumin** | >35 | 28-35 | <28 | g/l |
| **International Normalized Ratio** | <1.7 | 1.71-2.20 | > 2.20 | no units |
| **Ascites** | None | Suppressed with medication | Refractory | no units |
| **Hepatic**  **Encephalopathy** | None | Grade I-II  (or suppressed with medication) | Grade III-IV (or refractory) | no units |
