# Supplementary material for: A Canadian Critical Care Trials Group project in collaboration with the international forum for acute care trialists - Collaborative H1N1 Adjuvant Treatment pilot trial (CHAT): study protocol and design of a randomized controlled trial
Source: Trials. 2011 Mar 9;12:70. doi: 10.1186/1745-6215-12-70 (PMC3068961; doi:10.1186/1745-6215-12-70)
Supplement: Additional file 3 — Consent Algorithm (if Waiver of Consent Not Used). File containing the consent algorithm (if waiver of consent not used). [file 1745-6215-12-70-S3.DOC]

**Appendix 3: Consent Algorithm**
