# Supplementary material for: A Canadian Critical Care Trials Group project in collaboration with the international forum for acute care trialists - Collaborative H1N1 Adjuvant Treatment pilot trial (CHAT): study protocol and design of a randomized controlled trial
Source: Trials. 2011 Mar 9;12:70. doi: 10.1186/1745-6215-12-70 (PMC3068961; doi:10.1186/1745-6215-12-70)
Supplement: Additional file 4 — Illness Severity Assessment Scales. File containing the illness severity assessment scales. [file 1745-6215-12-70-S4.DOC]

**Appendix 4: Illness Severity Scale Assessment**

**(i) Multiple Organ Dysfunction Score** **(MODS)**

Instructions:

1. Use the first morning value for each variable recorded (around 08:00)

 a)  Day #1 = Day of randomization (baseline data)

b) Data to be calculated based on the calendar day

c) If missing data, either carry last observation forward (first choice) or score 0

d0 If the result for a specific test is not available, then a score of 0 is used for that test.

2. Non-survivors must be considered to have all organs failing on date of death.

3. The serum creatinine concentration is measured without reference to the use of dialysis.

4. The PO_2_ / FiO_2_ ratio (PO_2_ in mmHg and FiO_2_ in %) is calculated without reference to the use or mode of mechanical ventilation, and without reference to the use or level of positive end-expiratory pressure.

| **Multiple Organ Dysfunction Score (MODS)** | | | | | | |
| --- | --- | --- | --- | --- | --- | --- |
| Organ System Values | MODS Score | | | | | Normal Value Ranges |
|  | 0 | 1 | 2 | 3 | 4 |  |
| Haematologic: Platelet Count (x10^3^/mm^3^ or 10^9^/L) | > 120 | 81-120 | 51-80 | 21-50 | ≤ 20 | *> 120* |
| Hepatic: Serum Bilirubin (μmol/L) | ≤ 20 | 21-60 | 61-120 | 121-240 | > 240 | *≤ 20* |
| Renal: Serum Creatinine (μmol/L) | ≤ 100 | 101-200 | 201-350 | 351-500 | > 500 | *≤ 100* |
| Cardiovascular: PAR | ≤ 10 | 10.1-15 | 15.1-20 | 21-30 | > 30 | *≤ 10* |
| Glasgow Coma Score | 15 | 13-14 | 10-12 | 7-9 | ≤ 6 | *15* |
| Respiratory: PO_2_ / FiO_2_ | > 300 | 226-300 | 151-225 | 76-150 | ≤ 75 | *> 300* |

***Legend:***

CVP: central (or atrial) venous pressure (mmHg); GCS: Glasgow Coma Score; HR: heart rate (beats per minute); MAP: Mean Arterial Pressure (mmHg); ND: not done; PAR: Pressure Adjusted heat Rate (PAR; where PAR = HR x CVP*/MAP); HR: Heart Rate. Partial pressure of oxygen in arterial blood (PaO_2_); Fractional concentration of inspired oxygen (FiO_2_)

* If no CVP, set default value to 8 for calculation of PAR.

**(ii) Glasgow Coma Score (GCS)**

GCS is preferably calculated by the patient’s nurse, and is scored conservatively (for the patient receiving sedation or muscle relaxants, normal function is assumed, unless there is evidence of intrinsically altered mentation).

| **Glasgow Coma Scale** | | | | | | |
| --- | --- | --- | --- | --- | --- | --- |
|  | **1** | **2** | **3** | **4** | **5** | **6** |
| **Eyes** | Does not open eyes | Opens eyes in response to painful stimuli | Opens eyes in response to voice | Opens eyes spontaneously | N/A | N/A |
| **Verbal** | Makes no sounds | Incomprehensible sounds | Utters inappropriate words | Confused, disoriented | Oriented, converses normally | N/A |
| **Motor** | Makes no movements | Extension to painful stimuli | Abnormal flexion to painful stimuli | Flexion / Withdrawal to painful stimuli | Localizes painful stimuli | Obeys Commands |

The scale comprises three tests: eye, verbal and motor responses. The three values separately as well as their sum are considered. The lowest possible Glasgow Coma Scale (GCS) is 3 (deep coma or death), while the highest is 15 (fully awake person).

Intubation and severe facial/eye swelling or damage, make it impossible to test the verbal and eye responses. In these circumstances, the score is given as 1 with a modifier attached e.g. 'E1c' where 'c' = closed, or 'V1t' where t = tube. A composite might be 'GCS 5tc'. This would mean, for example, eyes closed because of swelling = 1, intubated = 1, leaving a motor score of 3 for 'abnormal flexion'

**(iii) Sequential Organ Failure Assessment Score (SOFA Score)**

| **Organ System** | **0** | **1** | **2** | **3** | **4** | **9** | **Organ Score** |
| --- | --- | --- | --- | --- | --- | --- | --- |
| **Respiratory**  **PaO_2_ / FiO_2_**  **(mmHg)** | > 400 | 301-400 | 201-300 (with or without respiratory support*)  <201 (without respiratory support*) | 101-200 (with respiratory support*) | ≤ 100 (with respiratory support*) | Variable not measured | _______ |
| **Coagulation**  **Platelets**  **(x 10^9^/L)** | > 150 | 101-150 | 51-100 | 21-50 | < 20 | Variable not measured | ________ |
| **Bilirubin**  **mg/dl**  **(OR µmol/L)** | < 1.2  < 20 | 1.2 – 1.9  20-32 | 2.0-5.9  33-101 | 6.0-11.9  102-204 | > 12.0  > 204 | Variable not measured | ________ |
| **Cardiovascular** | MAP>70  mmHg | MAP<70  mmHg | Dopamine≤5.0  (µg/kg/min)  or any dose of:  Dobutamine  Milrinone  Levosimendan | Dopamine 5.1-15.0  (µg/kg/min)  or Epinephrine≤0.1  or Norepinephrine≤0.1  or any dose of:  Vasopressin  Metaraminol  Phenylephrine | Dopamine > 15.0  (µg/kg/min)  or Epinephrine>0.1  or  Norepinephrine>0.1 | Variable not measured | _______ |
| **Renal creatinine**  **mg/dl**  **(OR µmol/L)**  **urine output** | < 1.2  < 110 | 1.2-1.9  110-170 | 2.0-3.4  171-299 | 3.5-4.9  300-440  or < 500 ml/day | > 5.0  > 440  or < 200 ml/day | Variable not measured | ________ |

***Respiratory support is defined as any form of invasive or noninvasive ventilation including mask CPAP or CPAP delivered through a tracheostomy/tracheotomy or endotracheal tube.**
