# Supplementary material for: A Canadian Critical Care Trials Group project in collaboration with the international forum for acute care trialists - Collaborative H1N1 Adjuvant Treatment pilot trial (CHAT): study protocol and design of a randomized controlled trial
Source: Trials. 2011 Mar 9;12:70. doi: 10.1186/1745-6215-12-70 (PMC3068961; doi:10.1186/1745-6215-12-70)
Supplement: Additional file 5 — Classification of Adverse Events. File containing the classification of adverse events. [file 1745-6215-12-70-S5.DOC]

**Appendix 5: Classification of Adverse Events**

We define a *reportable adverse event* as (i) any clinically important untoward medical occurrence in a patient receiving study drug or undergoing study procedures which is different from what is expected in the clinical course of a critically ill patient with influenza, or, (ii) any clinically important, untoward medical occurrence that is thought to be associated with the study drug or procedures, regardless of the “expectedness” of the event for the course of a patient with influenza.

***Expected Adverse Events***

Expected adverse events are untoward clinical occurrences that are perceived by the investigator to occur with reasonable frequency in the day to day care of mechanically ventilated patients with influenza treated in an ICU with/without mechanical ventilation. Adverse events that are expected in the course of this study may include (but are not limited to) transient hypoxemia, agitation, delirium, nosocomial infections, skin breakdown, venous thromboembolism and gastrointestinal bleeding. These events (often the focus of prevention efforts as part of usual care in the ICU) will not be considered reportable adverse events unless the event is considered by the investigator to be associated with the study drug or procedures, or unexpectedly severe or frequent for an individual patient with influenza. One example of an unexpected adverse event would be repeated episodes of unexplained hypoxemia (versus transient or isolated episodes of hypoxemia). Whereas the former is considered unexpected by nature, severity or frequency the latter is not.

***Serious, Unexpected, Study-related Adverse Events***

Investigators will report all events that are **serious** AND **unexpected** AND believed to be **study-related** to both the local REB and the Study Methods Centre by phone, fax or electronic mail within 24 hours of becoming aware of event. Investigators will submit a detailed written report to the Study Methods Center and REB no later than 5 calendar days after the investigator becomes aware of the event.

The Study Methods Centre will report all serious, unexpected, and study-related adverse events to the Study DSMB by electronic mail, or telephone, within 7 calendar days of the Study Methods Centre being notified of the event. A written report will be sent to the DSMB within 15 calendar days, and these reports will be sent to investigators for submission to their respective REBs. The DSMB will also review all adverse events during scheduled interim analyses.

The Study Methods Centre will also determine if the serious adverse event is unexpected for a statin. An unexpected event for a statin is defined as any event not listed in the rosuvastatin package insert. If the Study Methods Centre determines that any serious and study-related adverse event is unexpected for a statin, Health Canada will be notified within 7 calendar days.

***Unanticipated Problems***

Investigators must also report Unanticipated Problems, regardless of severity, associated with either study drug or study procedures within 24 hours. An unanticipated problem is defined as any incident, experience, or outcome that is:

(i) unexpected, in terms of nature, severity, or frequency, given the research procedures that are described in the protocol-related documents, such as the REB-approved research protocol and informed consent document; and the characteristics of the subject population being studied,

(ii) related or possibly related to participation in the research, in this guidance document, possibly related means there is a reasonable possibility that the incident, experience, or outcome may have been caused by the procedures involved in the research,

(iii) suggests that the research places subjects or others at a greater risk of harm (including physical, psychological, economic, or social harm) than was previously known or recognized.
