# Supplementary material for: A Canadian Critical Care Trials Group project in collaboration with the international forum for acute care trialists - Collaborative H1N1 Adjuvant Treatment pilot trial (CHAT): study protocol and design of a randomized controlled trial
Source: Trials. 2011 Mar 9;12:70. doi: 10.1186/1745-6215-12-70 (PMC3068961; doi:10.1186/1745-6215-12-70)
Supplement: Additional file 6 — Special Considerations. File containing special considerations. [file 1745-6215-12-70-S6.DOC]

**Appendix 6: Special Considerations**

***Patient Withdrawals***

Patients will have the right to withdraw from the study at any time. In both groups, patients will be permitted to suspend the assigned treatment protocol if the patient/SDM requests study withdrawal for patients enrolled under a waiver of consent. Additionally, clinicians may suspend the assigned treatment protocol in the event of concerns regarding hepatic toxicity or in the presence of a Creatinine Clearance < 10 mls/min or the requirement for dialysis (hemodialysis, continuous renal replacement therapy, sustained low efficiency dialysis, peritoneal dialysis). These circumstances will not be considered as study withdrawals.

Under the rare circumstance that a patient/SDM withdraws the patient from the study, we will include data collected up to the time of withdrawal unless approval to continue to collect data from registries or databases is obtained. If a patient/SDM also withdraws his/her permission to have his/her/the patient’s data included up to that point, the data will be removed from the dataset. We will make every effort to encourage patients to allow us to include their data and conduct the planned ITT analysis. We will document study withdrawals, cross-overs and permission/refusal to include data by extensive documentation in the patient’s chart and on the case report forms.

***Consent***

**During a pandemic, there is a need for researchers and REBs to find balance between the ethical principle of autonomy and the individual right to information privacy with social justice and population ethics.** Relying exclusively on *a priori* surrogate consent for study enrolment during the current H1N1 influenza pandemic may preclude the conduct of clinical research and yield the following undesirable outcomes: slow study enrolment, denial of the opportunity to benefit from research participation, limitation of the generalizability of study findings, delay in the identification of treatments as effective, ineffective, or harmful, and lack of acquisition of new knowledge about the pandemic.

**Hybrid consent models (e.g., different consent models within the same study) that acknowledge whether surrogates exist or are unavailable should be encouraged, adapted to local norms and circumstances. Verbal consent should be considered for surrogates who are illiterate. Consent through translators should be considered for surrogates speaking different languages or dialects. Telephone consent should be considered for surrogates who are prohibited from or are unable to come to hospital during a pandemic.**

**Regardless of the consent model utilized, we intend to collect study data on all randomized patients in order to limit the** potential for selection bias. Including data from survivors only may result in biased estimates of treatment effect. We anticipate these circumstances will be infrequent and have outlined a clear plan for data management under a deferred consent paradigm *a priori*. We will document the number of study withdrawals, reason for withdrawal and permission/refusal to include data in the study analysis.

In Article 2.8, the TriCouncil Policy Statement (TCPS) [38] sets forth several conditions for research in emergency health situations. The REB may allow research that involves health emergencies to be carried out without the free and informed consent (i.e., a waiver of consent) of the subject or of his or her authorized third party if ALL of the following apply:

- A serious threat to the prospective subject requires immediate intervention

Experience with acute respiratory failure in general and H1N1 influenza specifically

In the ICU suggests that it is important to commence anti-inflammatory therapy as early as possible.

- Either no standard efficacious care exists or the research offers a real possibility of direct benefit to the subject in comparison with standard care

There are many questions about the optimal therapy for patients with H1N1 associated critical illness. There is clinical equipose on the use of statins in patients with H1N1. Data from experimental and animal models, and emerging human data, suggests that statins can attenuate inflammation. Morbidity and mortality in sepsis result from an uncontrolled inflammation response. If statins can mediate this response, they may confer benefit to critically ill patients with influenza infection.

- Either the risk of harm is not greater than that involved in standard efficacious care, or it

Is clearly justified by the direct benefits to the subject

There are minimal additional risks associated with the administration of rosuvastatin.

- The prospective subject is unconscious or lacks capacity to understand risks, methods

and purposes of the research

The patients who will be eligible to participate in this study will be critically ill. As a result

of their illness and requisite interventions such as mechanical ventilation and sedation,

they will likely lack decision-making capacity and thus be unable to participate in the

informed consent process.

- Third-party authorization cannot be secured in sufficient time, despite diligent and

documented efforts to do so.

Relying exclusively on a priori patient or surrogate consent for this study may preclude our ability to enroll patients in this study in a timely fashion. If projections are accurate and there are numerous patients fitting the study criteria in the ICU at any given time, it will be nearly impossible, given existing and anticipated resources, to expend the time necessary to secure *a priori* surrogate consent. Relying exclusively on a priori patient or surrogate consent for this study may preclude our ability to enroll patients in this study in a timely fashion.

- No relevant prior directive by the subject is known to exist.

Very few patients are admitted to the ICU with an advanced directive related to research.

*Patients Enrolled Under Waiver of Consent Model*

At sites where the local REB approves participant enrolment under a waiver of consent, research coordinators will present family members (namely the SDM) with an information letter outlining the rationale for the study, the study interventions, the risks and benefits of participation, privacy and confidentiality, and the right to withdraw from further participation. SDMs will be invited to ask questions and will be provided with contact numbers for research staff in the event that they have any further questions or concerns. Research coordinators will repeat this process with participants if they regain capacity during their ICU stay.

*Patients Enrolled Under Deferred Consent Model*

At sites where the local REB approves participant enrolment under a deferred consent model, research staff will make regular efforts to obtain first party consent/assent as long as the patient is (i) judged capable of providing consent and (ii) remains in the ICU. For patients lacking decision-making capacity, attempts will be made to identify and locate an SDM and seek consent soon as possible after randomization. SDMs will be invited to ask questions and will be provided with contact numbers for research staff in the event that they have any further questions or concerns. Research coordinators will repeat this process with participants if they regain capacity during their ICU stay.

***Eligible, Non-randomized Patients***

Eligible but non-randomized patients will be identified through the use of study screening logs. We will request a minimum dataset on all eligible but non-randomized patients for the purpose of comparing their demographic data and outcomes to those of the two treatment groups in the proposed trial according to local REB norms. This information will be de-identified (gathered using only the screening log reference number). Since each patient will be assigned a unique identifier, we will be able to verify the number of eligible but not randomized patients by cross-referencing assigned numbers in this study with those in the ongoing Influenza A H1N1 (Swine Flu) ICU Study

***Equitable Selection of Subjects***

A dedicated research coordinator will screen patients in the ICU to determine if any meet the study inclusion. No protocol-specific tests or procedures will be performed as part of the screening process. Justifications for exclusion criteria are provided in Section 5.4. Consistent with the principle of distributive justice, these exclusion criteria neither unjustly exclude nor unjustly include selected individuals from participation in the trial. A hybrid approach to consent, inclusive of the options of a waiver of consent and deferred consent, is proposed to ensure that vulnerable subjects (specifically incapable patients without SDMs) are not routinely excluded from the opportunity to participate.

***Justification for Including Vulnerable Subjects***

The present research aims to investigate the safety and efficacy of adjuvant treatment with rosuvastatin for mechanically ventilated patients admitted to the ICU with suspected, probably or confirmed H1N1 infection. Potential benefits to participation in this study are increased survival and reduced time on mechanical ventilation.

***Women of Childbearing Age***

## A pregnancy test result for women of childbearing potential must be available from this hospitalization through routine care or the patient should not be enrolled due to inability to determine pregnancy status.

***Justification for Excluding Pregnant Women***

A paucity of information regarding the safety of rosuvastatin exists in pregnant women. For this reason, pregnant women will be excluding from the CHAT Pilot trial.

**Oversight**

These studies will be conducted in accordance with the ethical principles stated in the Declaration of Helsinki (2008). These studies will be performed in accordance with the International Conference on Harmonization Guidelines- Good Clinical Practice and the TCPS statement for the Ethical Conduct of Research Involving Humans.

***Data Safety Monitoring Board***

An independent DSMB will play a key role in ensuring the continued safety of patients in this trial. The DSMB will consist of 3 individuals, one from outside Canada, with experience in clinical trials, critical care, and biostatistics. The DSMB will evaluate the feasibility data from the Pilot Trial and provide guidance on study continuation to the Steering Committee. The DSMB will evaluate all serious unexpected adverse drug reactions and will meet to review the full trial data at interim analyses scheduled to take place after 350 and 700 patients are enrolled and thereafter at the discretion of the DSMB. A DSMB charter will be created to document the terms of reference and procedures which will outline their specific responsibilities for the trial.
