# Supplementary material for: A Canadian Critical Care Trials Group project in collaboration with the international forum for acute care trialists - Collaborative H1N1 Adjuvant Treatment pilot trial (CHAT): study protocol and design of a randomized controlled trial
Source: Trials. 2011 Mar 9;12:70. doi: 10.1186/1745-6215-12-70 (PMC3068961; doi:10.1186/1745-6215-12-70)
Supplement: Additional file 7 — Letter of Information. File containing the letter of information. [file 1745-6215-12-70-S7.DOC]

**Appendix 7: Letter of Information**

**Letter of Information and Consent Form to**

**Participate in a Research Study**

**Study Title: Collaborative H1N1 Adjuvant Treatment (CHAT) Pilot Trial**

**Purpose of the Research:**

This study is being conducted to collect information on individuals with influenza infection (flu).

This year, we have seen an outbreak of very severe disease in Canada where some people with H1N1, otherwise known as “swine flu”, have developed life threatening illness with breathing failure requiring treatment in an intensive care unit (ICU). These patients have required the support of a mechanical ventilator (breathing machine) as a result of their infection. Part of the problem with infections like influenza is the excess inflammation that they cause in the body. Inflammation causes lung injury and may cause the lungs to fill up with fluid making it difficult to breath. The purpose of this study is to evaluate the role of Rosuvastatin (a cholesterol-lowering medication with anti-inflammatory effects) in reducing the amount of time that patients require life support therapy on a breathing machine. Rosuvastatin is not a new medicine. It is approved for sale in Canada and in many other countries. Rosuvastatin is commonly used in out-patients to reduce levels of cholesterol in the blood; however it has never previously been tested in patients with acute respiratory failure and influenza in the ICU. Health Canada has permitted the conduct of this study amongst patients with influenza infection [pending].

## Description of the Research:

If you agree and consent to participate in this study, you will be randomly assigned (like the flip of a coin) to one of 2 different groups:

*Group 1:*

Rosuvastatin (active medication by mouth or feeding tube)

*Group 2:*

Placebo (inactive powder by mouth or feeding tube)

If you participate in the study, you have a 50% chance of being in one of these groups. Neither you, nor the research study staff, nor the doctors can influence or know which group you are in. You will receive the study medications for up to 14 days. Rosuvastatin or placebo will be administered once daily as a crushed powder via a feeding tube or by mouth. Your participation in the study will last approximately 90 days. Approximately 80 individuals will be enrolled in the pilot study from hospitals across Canada.

**Potential Harms (Injury, Discomforts, Inconvenience):**

It is possible that Rosuvastatin could injure your liver and/or your muscles. Other symptoms you may have while taking Rosuvastatin include muscle or joint pain, headache, dizziness, nausea, abdominal pain, constipation, joint pain, weakness. Less than 2% of patients taking Rosuvastatin have other symptoms like blood in the urine, increased blood sugar, allergic reactions such as hives, rash, and itching, some loss of memory, inflammation of the pancreas or kidney damage.

You may have a have a lower, higher, or the same risk of dying from your illness than if you were not in the study. You may use the breathing machine for a shorter, longer, or the same amount of time than if you were not in the study.

## Potential Benefits:

There may or not be direct benefit to you from participating in this study. If you receive Rosuvastatin, it is possible that your illness may be less severe and/or you may improve more quickly. With the global emergence of H1N1 influenza infection, the results of this study will assist researchers to better understand influenza and to design treatments for future patients with the disease.

## Protecting Your Personal Health Information:

All persons involved in the study, including the study investigators and coordinators (hereby referred to as “study staff”), the study sponsor (Dr. John Marshall), are committed to respecting your privacy. No other persons will have access to your personal health information without your consent, unless required by law.

## Participation & Withdrawal:

Your participation in this study is entirely voluntary. **If you choose not to participate, you and your family will continue to have access to customary care at St. Michael’s Hospital. If you choose to take part in this study, you can withdraw from the study at any time without any effect on the care you or your family will receive at St. Michael’s Hospital by notifying the study staff.**

**Further Information and Study Contact:**

If you have any further questions about this study, please contact Dr. Marshall at (416) 864-6060 Ext. 5225 Monday through Friday, 9 a.m. to 5 p.m. If you have questions at any other time, you may page Orla Smith, RN at (416) 685-9902.
