# Supplementary material for: A Canadian Critical Care Trials Group project in collaboration with the international forum for acute care trialists - Collaborative H1N1 Adjuvant Treatment pilot trial (CHAT): study protocol and design of a randomized controlled trial
Source: Trials. 2011 Mar 9;12:70. doi: 10.1186/1745-6215-12-70 (PMC3068961; doi:10.1186/1745-6215-12-70)
Supplement: Additional file 8 — Telephone Consent. File containing telephone consent. [file 1745-6215-12-70-S8.DOC]

**Appendix 8: Telephone Consent**

**(i) Script for Telephone Consent**

The following general statements may be used in the initial telephone conversation with substitute decision makers (if required). Please note that it is difficult to script the conversation given that it is not possible to anticipate the responses of the SDM with any degree of certainty.

*Hello (insert name of SDM). My name is (insert name of research personnel) and I am the research nurse/coordinator in the (insert name of unit). As a teaching and research facility, (insert name of hospital) participates in a number of research studies. In critical care, research is the best method we have to advance our understanding of disease and improve detection, prevention, and treatment of critical illness. As such, we feel it is important to offer opportunities for research participation to our patients and families for their consideration. When patients are in the ICU, we are usually not able to converse with them due to the severity of their illness or the treatments that we administer to support them (mechanical ventilation, sedation). Because of this, we ask family members to act as substitute decision makers and to make decisions for the patient based on their best knowledge of what the patient would want for themselves if they could speak. Participation in any type of research is entirely voluntary and you have the right to refuse research or withdraw from research at any time.*

*As (insert name of physician) has explained to you, (insert patient name) has been diagnosed with (suspected/probable/confirmed) H1N1 influenza infection. We are currently conducting a cross-Canada study to better understand how to treat patients with H1N1 illness in the ICU. This study involves the use of a medication called rosuvastatin. Rosuvastatin is a medication with anti-inflammatory effects that is commonly used to manage cholesterol. Would you be willing to discuss the possibility of (insert name of patient) participating in a research study with me?*

If the SDM is willing to discuss the possibility of research participation, we would then review the contents of the consent form. We would also send a copy of the consent form via fax or email (if possible) to assist the SDM in the decision making process. We would endeavor to have the SDM sign the original consent document at their earliest convenience if possible (i.e. first visit to the hospital).

Please note that we document our consent discussion both in our study file and in the patient’s standing medical chart. Please also note that we would ensure the presence of a witness to the conversation to attest to the content of the discussion if occurring via telephone. The Telephone Consent Questionnaire would also be administered and completed.

**(ii) Telephone Consent Questionnaire**

Did the substitute decision-maker receive a copy of the consent form before or during the telephone conversation?

Yes  No  f yes, was the form sent by: Fax  Email

|  | | **Clear** | | **Re-explained** |
| --- | --- | --- | --- | --- |
|  | | **Yes** | **No** | **Yes** |
| **Voluntary** | |  |  |  |
| 1a. | Do you have to agree to the patient’s participation in the research study? |  |  |  |
| 2. | Once you have signed the consent form, does the patient have to stay in the research study until the end? |  |  |  |
| 3. | If you decide not to consent to the study, will the way your doctors and nurses feel about you or the patient change in any way? |  |  |  |
| **About the Research Study** | |  |  |  |
| 4. | What is the purpose of the study? |  |  |  |
| **Risk and Benefits** | |  |  |  |
| 5. | What are the benefits of being in the study for the patient? |  |  |  |
| 6. | What are the risks of being in the study for the patient? |  |  |  |
| **Confidentiality** | |  |  |  |
| 7. | Will the patient’s study files be kept confidential? |  |  |  |
| **Time Required** | |  |  |  |
| 8. | How long will the patient be required to participate in this study? |  |  |  |
| **Reimbursement** | |  |  |  |
| 9. | Will you or the patient be paid for taking part in this study? |  |  |  |
| **Questions** | |  |  |  |
| 10. | If you have specific questions about this study, who should you ask? |  |  |  |
| 11. | If you have questions about being involved in a research study in general, who should you ask? |  |  |  |

**CONSENT STATEMENT** I have explained to the above Substitute Decision Maker (SDM) the nature and purpose, the potential benefits, and possible risks associated with the patient’s participation in this research study. I have answered all questions that have been raised by the SDM.

______________________ _______________________ __________________

Printed name of Person Conducting Signature of Person Conducting Date and Time (24h clock)

Consent & Position Consent

Impartial witness Statement I confirm that the information in the consent summary and any other written information was accurately explained to, and apparently understood by, the Participant. The Participant freely consented to participate in the research study.

____________________________ ___________________ ___________________

Printed Name of Witness Signature of Witness Date and Time (24h clock)

Completed By: _________________ Signature _______________ Date: ___________________

Copy of this signed document given to SDM in person ____________________

Date and Time (24 h clock)

Copy of this signed document faxed to SDM via secure fax ____________________

Date and Time (24 h clock)
